# Identification of the major fermentation inhibitors of recombinant 2G yeasts in diverse lignocellulose hydrolysates

**DOI:** 10.1186/s13068-021-01935-9

**Published:** 2021-04-09

**Authors:** Gert Vanmarcke, Mekonnen M. Demeke, Maria R. Foulquié-Moreno, Johan M. Thevelein

**Affiliations:** 1 Laboratory of Molecular Cell Biology, Institute of Botany and Microbiology, Department of Biology, KU Leuven, Kasteelpark Arenberg 31, 3001 Leuven-Heverlee, Flanders Belgium; 2 Center for Microbiology, VIB, Kasteelpark Arenberg 31, 3001 Leuven-Heverlee, Flanders Belgium; 3 NovelYeast bv, Open Bio-Incubator, Erasmus High School, Laarbeeklaan 121, Jette, 1090 Brussels, Belgium

**Keywords:** Bioethanol production, Lignocellulose hydrolysates, Fermentation inhibitors, 2G yeast strains

## Abstract

**Background:**

Presence of inhibitory chemicals in lignocellulose hydrolysates is a major hurdle for production of second-generation bioethanol. Especially cheaper pre-treatment methods that ensure an economical viable production process generate high levels of these inhibitory chemicals. The effect of several of these inhibitors has been extensively studied with non-xylose-fermenting laboratory strains, in synthetic media, and usually as single inhibitors, or with inhibitor concentrations much higher than those found in lignocellulose hydrolysates. However, the relevance of individual inhibitors in inhibitor-rich lignocellulose hydrolysates has remained unclear.

**Results:**

The relative importance for inhibition of ethanol fermentation by two industrial second-generation yeast strains in five lignocellulose hydrolysates, from bagasse, corn cobs and spruce, has now been investigated by spiking higher concentrations of each compound in a concentration range relevant for industrial hydrolysates. The strongest inhibition was observed with industrially relevant concentrations of furfural causing partial inhibition of both D-glucose and D-xylose consumption. Addition of 3 or 6 g/L furfural strongly reduced the ethanol titer obtained with strain MD4 in all hydrolysates evaluated, in a range of 34 to 51% and of 77 to 86%, respectively. This was followed by 5-hydroxymethylfurfural, acetic acid and formic acid, for which in general, industrially relevant concentrations caused partial inhibition of D-xylose fermentation. On the other hand, spiking with levulinic acid, 4-hydroxybenzaldehyde, 4-hydroxybenzoic acid or vanillin caused little inhibition compared to unspiked hydrolysate. The further evolved MD4 strain generally showed superior performance compared to the previously developed strain GSE16-T18.

**Conclusion:**

The results highlight the importance of individual inhibitor evaluation in a medium containing a genuine mix of inhibitors as well as the ethanol that is produced by the fermentation. They also highlight the potential of increasing yeast inhibitor tolerance for improving industrial process economics.

**Supplementary Information:**

The online version contains supplementary material available at 10.1186/s13068-021-01935-9.

## Background

Second-generation (2G) bioethanol has been proposed as an attractive, alternative fuel in the transportation sector [1, 2]. From the abundantly available lignocellulosic biomass (waste/side streams and energy crops), other value-added chemicals can also be produced (e.g. building blocks for bioplastics) [3, 4]. With respect to the fermentation part of the industrial process, two crucial challenges have to be overcome to make industrial production economically viable. The first is efficient fermentation of the pentose sugar, xylose, which can constitute up to 35% of the fermentable sugar in lignocellulosic biomass [5, 6]. This challenge has been addressed by expressing heterologous genes in the yeast *Saccharomyces cerevisiae*, which establish an efficient fungal-derived xylose reductase/xylitol dehydrogenase or bacterial derived xylose isomerase pathway. Although both pathways can support efficient xylose fermentation, redox imbalance in the first pathway can lead to excessive xylitol production especially in real lignocellulose hydrolysates under industrial fermentation conditions. In spite of this, combining both pathways in a single yeast strain might be beneficial [7]. On the other hand, the artificially engineered capacity of xylose fermentation has turned out to be much more sensitive to the high levels of toxic chemicals present in lignocellulose hydrolysates [8–14], which effectively makes completion of xylose fermentation the rate limiting part in the whole fermentation process of lignocellulose hydrolysates.

The second main challenge for efficient conversion of lignocellulose hydrolysates, i.e. the presence of high levels of inhibitory chemicals has now taken center stage [15, 16]. Although methods have been developed for detoxification of lignocellulose hydrolysates and mild pre-treatment technologies have been devised that generate only low levels of inhibitors [12, 15, 17], these technologies all add significant additional cost to the industrial process, making the achievement of economic viability even more challenging. In general, the harsh but cheap pre-treatment technologies generate much higher levels of toxic chemicals than the more expensive, mild technologies [15, 18, 19], which underscores the importance of yeast inhibitor tolerance for reaching economic viability in fermentation processes with lignocellulosic biomass [20].

The inhibitory compounds that are generated during pre-treatment of lignocellulosic biomass severely affect yeast fermentation rate and yield [11, 12, 21, 22]. They include weak acids (e.g. acetic acid, formic acid and levulinic acid), furan aldehydes (e.g. furfural and 5-hydroxymethylfurfural (HMF)), and phenolic compounds (e.g. 4-hydroxy-3-methoxybenzaldehyde or vanillin, 4-hydroxybenzoic acid, 4-hydroxybenzaldehyde, coniferyl alcohol, 4-hydroxy-3-methoxycinnamaldehyde, coniferyl aldehyde and *p*-coumaric acid). Furfural is present in lignocellulose hydrolysates as a breakdown product of pentose (C5) sugars, and HMF as that of hexose (C6) sugars. Prolonged pre-treatment or increased severity of the pre-treatment method, results in degradation of these compounds to formic acid and levulinic acid [21]. Acetic acid is released during pre-treatment upon deacetylation of the hemicellulose fraction, while phenolic compounds are released during partial breakdown of lignin. Although many reports have described the inhibitory effects of these compounds on the fermentation capacity of *Saccharomyces cerevisiae* strains, these studies were mostly carried out with laboratory strains, synthetic media, single inhibitors or with inhibitor concentrations much higher than those generally found in lignocellulose hydrolysates [10, 23, 24]. More recently, attention has focused on using more real-life 2G bioethanol production conditions, by developing 2G industrial yeast strains and evaluating their performance in lignocellulose hydrolysates [25, 26]. Yeast fermentations in lignocellulose hydrolysates imply inhibition by a mixed cocktail of all inhibitory compounds present as well as the ethanol that is produced during the fermentation [12]. However, the composition and especially the relevance of the different toxic compounds present in the mixture has remained unknown [27].

The inhibitor profile present in lignocellulose hydrolysates varies widely and strongly depends on the type of biomass and pre-treatment technology used (Table 1) [16, 28]. Waste material and side streams of different origins have been used, such as wheat straw, barley straw, corn stover, forestry residues of pine and birch, hardwood chips, biowaste, cardboard, rice husks and different types of bagasse. A variety of technologies for pre-treatment of these materials has been applied. They include physico-chemical pre-treatment (e.g. steam explosion) and dilute acid treatment (e.g. sulphuric acid) [17, 21, 29–34]. For example, corn stover pre-treated in different ways resulted in hydrolysates with a wide range of inhibitor concentrations (i.e. 1.6 to 5.0 g/L acetic acid, 0.1 to 0.7 g/L HMF and 0.3 to 8.5 g/L furfural) [35]. Also, pre-treatment of agave bagasse with variable temperature or duration, and a varying HCl or H_2_SO_4_ concentration resulted in widely different sugar yields and inhibitor concentrations (3.7 to 10.3 g/L acetic acid, 0.3 to 7.4 g/L formic acid). The highest sugar yields obtained with HCl or H_2_SO_4_ pre-treatment also resulted in one of the highest inhibitor loads observed, i.e. 2.0 or 3.6 g/L formic acid and 8.3 or 7.8 g/L acetic acid, respectively [30].

**Table 1:** Inhibitor composition of different lignocellulose hydrolysates as reported in the literature

Hydrolysate	Acetic Acid (g/L)	Formic Acid (g/L)	Levulinic acid (g/L)	Furfural (g/L)	HMF (g/L)	References
Agave bagasse	7.8–8.3	2.0–3.6	N.D	N.D	N.D	[30]
Alder	9.1–11.2	N.D	N.D	0.2–1.4	2.6–4.5	[34]
Aspen hardwood	8.2–10.1	0.0	0.0	2.1–3.5	1.3–6.8	[34]
Bark	0.0–6.2	N.D	N.D	0.5–1.0	0.4–4.3	[34]
Barley straw	N.D	N.D	N.D	2.9	1.0	[32]
Biowaste	2.0	N.D	N.D	0.0	N.D	[29]
Birch hardwood	2.0–11.5	4.6	0.0	0.2–4.6	0.1–5.8	[29, 34, 53]
Cardboard	0.5	N.D	N.D	0.1	N.D	[29]
Coffee husks	2.9	N.D	N.D	0.0	0.3	[54]
Corn cob	6.0	N.D	N.D	0.4	N.D	[29]
Corn stover	0.0–2.2	0.0–6.8	0.0–2.2	0.6–11.0	0.1–5.3	[35, 55]
Oil palm	5.0–9.0	N.D	N.D	1.0–1.2	N.D	[29]
Pine	0.0–3.7	0.0	0.0	0.7–6.9	1.0–8.6	[34]
Rice husks	1.8–2.1	2.5–2.7	N.D	N.D	N.D	[30]
Rice straw	2.3	N.D	N.D	0.1	0.3	[33]
Sorghum bagasse	1.0	0.16	0.2	0.0	1.6	[31]
Spruce	0.0.–4.7	0.6–3.1	0.2–3.2	0.2–1.4	0.5–8.4	[17, 21, 32, 34, 56]
Sugar cane bagasse	N.D.–4.9	N.D.–2.5	N.D.–2.7	0.1–3.1	0.1–3.0	[17, 29, 54]
Wheat straw	3.0–7.0	0.0–1.3	0.0	0.4–1.4	0.1–0.3	[29, 32, 40]
Willow	N.D	N.D	N.D	0.3–3.2	0.6–3.9	[34]

The hydrolysates listed were made with different types of biomass and different pre-treatment methods, resulting in broad variation in the levels of some inhibitors. The level of phenolic compounds was only determined in a limited number of studies and these data are not included in the table

*ND *not determined, *EFB* empty fruit bunch

Acetic acid concentrations in lignocellulose hydrolysates generally are most abundant compared to other inhibitors and often range from 0.4% to 0.6% (v/v), followed by furfural, HMF, formic acid and levulinic acid. Synergistic effects between these inhibitors can strongly enhance hydrolysate toxicity and severely reduce fermentation rate and yield of 2G bioethanol yeasts [11]. Many lignin-derived phenolics are also present in hydrolysates but concentrations of these compounds are generally 10 to 100 times lower compared to weak acids and furan aldehydes. Nevertheless, small amounts of these phenolics can sometimes exert potent inhibition of the fermentation capacity of 2G yeasts [36].

In this work, the relevance of the main chemical inhibitors in their native lignocellulose hydrolysate fermentation environment has been investigated by spiking the hydrolysates with different concentrations of the chemicals, and therefore taking into account the whole mixture of additional inhibitors present in their native concentrations as well as the ethanol produced during the fermentation. This was performed with five different biomass hydrolysates and with two industrial 2G yeast strains with a distinct state of development for improvement of inhibitor tolerance.

## Results and discussion

To identify the major fermentation inhibitors for 2G bioethanol production present in five lignocellulose hydrolysates from different biomass origin, the fermentation performance of industrial yeast strains GSE16-T18 and MD4 was assessed in the hydrolysates spiked with different concentrations of the inhibitors (Table 2). The spiked concentrations were adjusted to the inhibitor concentration already present and chosen so as to cover the concentration range of the inhibitors reported in the literature (Tables 1, 2). The hydrolysates used were two sugarcane bagasse hydrolysates (BH 31B and BH 18), two corn cob hydrolysates (CCH 22 and CCH 31A) and one spruce hydrolysate (SH 1), of which the composition is shown in Table 2.

**Table 2:** Composition of the lignocellulose hydrolysates used in this study and the level of spiked inhibitors

Hydrolysate Component	Bagasse BH 31B (% w/v)	Bagasse BH 18 (% w/v)	Corn cobs CCH 31A (% w/v)	Corn cobs CCH 22 (% w/v)	Spruce SH 1 (% w/v)	Inhibitor Concentration range from literature (% w/v)	Inhibitor concentrations added to each hydrolysate (% w/v)
Glucose	6.12	6.20	6.88	6.50	4.2	N.A	N.A
Xylose	3.92	4.30	5.66	4.00	1.3	N.A	N.A
Arabinose	0.20	0.27	0.47	0.40	0.32	N.A	N.A
Acetic Acid	0.53	0.43	0.63	0.60	0.70	0.10–1.10	0.03, 0.06, 0.10, 0.30, 0.60, 0.80
Levulinic Acid	0.01	0.00	0.00	0.00	0.17	0.00–0.32	0.03, 0.06, 0.10, 0.30, 0.60
Formic Acid	0.06	0.03	0.03	0.03	0.03	0.00–0.68	0.03, 0.06, 0.10, 0.30, 0.60
Furfural	0.03	0.03	0.04	0.02	0.27	0.02–0.35 *	0.03, 0.06, 0.10, 0.30, 0.60
HMF	0.01	0.02	0.01	0.02	0.53	0.01–0.59**	0.03, 0.06, 0.10, 0.30, 0.60
4-hydroxy benzoic acid	N.D	N.D	N.D	N.D	N.D	0.0000–0.0011	0.0001, 0.0003, 0.0010
4-hydroxy benzaldehyde	N.D	N.D	N.D	N.D	N.D	0.0000–0.0110	0.0003, 0.0010, 0.0030
Vanillin	N.D	N.D	N.D	N.D	N.D	0.000–0.041	0.003, 0.010, 0.030

The concentration of sugars and inhibitors present in the hydrolysates, as well as the inhibitor concentrations spiked, are indicated. The inhibitor concentration range described in the literature for weak acids and furan aldehydes is derived from the data collected in Table 1 and for the phenolic compounds based on other literature references [40, 57, 58]

*NA* not applicable, *ND* not determined, *BH* bagasse hydrolysate, *CCH* corn cob hydrolysate, *SH* spruce hydrolysate

*Concentrations of furfural present in lignocellulose hydrolysates were found to be up to 0.35% (w/v) (Table 1), with exception of corn stover hydrolysates in two studies (up to 0.85% (w/v) [35] and 1.10% (w/v) furfural [55])

**HMF concentrations present in different lignocellulose hydrolysates were found to be up to 0.59% (w/v) (Table 1), with exception of hydrolysates from woody energy crops pre-treated at high temperature (at least 222 °C) (up to 0.86%, w/v, HMF). The same biomasses pre-treated at lower temperatures contained HMF concentrations below 0.59% (w/v) [34]

Two different 2G yeast strains with xylose utilizing capacity, GSE16-T18 (diploid) and MD4 (tetraploid), were used. They were developed from the strain GS1.11–26 [37] using evolutionary engineering or genome shuffling for higher inhibitor tolerance and performance in lignocellulose hydrolysates (see Materials and Methods). It is important to assess inhibitor tolerance with recombinant xylose utilizing yeast strains since the artificially engineered xylose fermentation capacity was found to be most sensitive to inhibitors and therefore constitutes the main limiting factor for reaching adequate levels of yield, productivity and product titer in 2G bioethanol fermentations [8–11, 13, 14].

### Evaluation of the second-generation yeast strains for fermentation capacity in different lignocellulose hydrolysates

**Fig. 1:**
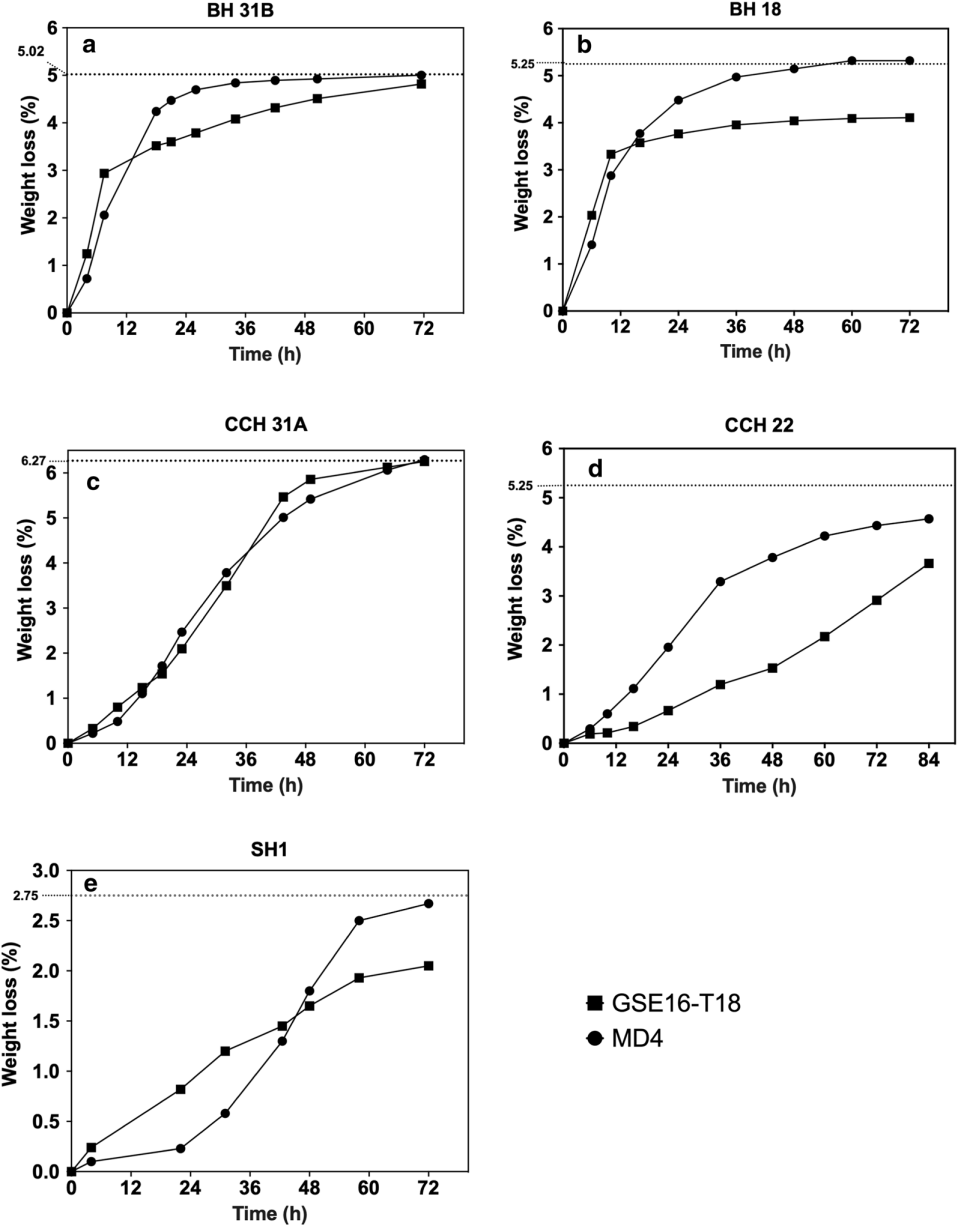
Fermentation performance of 2G yeast strains in different lignocellulose hydrolysates. Small-scale (10 mL) fermentations with GSE16-T18 and MD4 at pH 5.2, 35 °C, 350 rpm, initial OD_600_ of 5.0 in **a** Bagasse BH 31B, **b** Bagasse BH 18, **c** Corn cobs CCH 31A, **d** Corn cobs CCH 22, and **e** Spruce SH 1. Maximum weight loss expected upon complete glucose and xylose consumption is indicated by the dashed line

First, the fermentation performance of GSE16-T18 and MD4 was evaluated in small-scale fermentations with different unspiked lignocellulose hydrolysates. Weight loss was determined to follow the progress of the fermentation. In bagasse hydrolysate 31B (BH 31B), glucose and xylose fermentation was largely completed after 36 h by strain MD4, while strain GSE16-T18 needed about 72 h to complete the fermentation (Fig. 1a). HPLC analysis revealed that the initial fermentation rate with glucose was similar for the two strains, confirming that efficient xylose fermentation is a major obstacle for bioethanol production with lignocellulose hydrolysates. A similar difference between the two strains was seen with the second bagasse hydrolysate (BH18), which appeared to be more toxic causing poor fermentation by GSE16-T18 in the second, xylose utilization phase (Fig. 1b). The fermentation of the corn cob hydrolysates, CCH 31A and CCH 22, was much slower than that of the bagasse hydrolysates, but only in the CCH22 hydrolysate MD4 displayed a better fermentation than GSE16-T18 (Fig. 1c, d). In the spruce hydrolysate SH1, GSE16-T18 initiated glucose fermentation faster, but MD4 again showed a better completion of the xylose utilization in the second phase (Fig. 1e). Hence, in all hydrolysates, except for CCH 31A, MD4 showed superior xylose fermentation capacity. This is also reflected in the final ethanol titer obtained after 72 h of fermentation (Additional file 1: Tables S1–S4), indicating a higher fermentation efficiency (ratio of ethanol titer/initial glucose and xylose content) of MD4 in all hydrolysates, except in corn cob CCH 31A hydrolysate where both strains reached 100% efficiency. On the other hand, there is still need for further improvement of the fermentation capacity of MD4 with some types of biomass hydrolysates, especially corn cob CCH2 hydrolysate.

The difference in fermentability between the hydrolysates is likely due to variation in the total inhibitor concentration as well as the inhibitor profile. Based on our data, with exception of CCH 22, a correlation appears to exist between the toxicity of a hydrolysate and its acetic acid concentration or total inhibitor load present. In a previous study, the rape straw hydrolysate appeared to be much less toxic for the yeast strain evaluated [26]. This could be explained by a lower acetic acid concentration (3.84 g/L) and total combined inhibitor load (5.55 g/L) compared to all hydrolysates evaluated in this study, with exception of BH 18. In addition, total furfural content and total furan aldehyde content have been described as predictors of fermentation rate in lignocellulose hydrolysates [34, 38]. To a certain extent, indeed, in this study, the fermentation rate was similar in unspiked BH 31B, BH 18 and CCH 31A hydrolysates, that all contain about the same amount of furan aldehydes. Their level is much higher in SH 1, which also shows a much slower fermentation rate. Also, in CCH 22 hydrolysate, a higher inhibition of both yeast strains was observed. This could be due to other inhibitory compounds present, such as phenolics, of which the concentration was not assessed in our work.

### Evaluation of second-generation yeast fermentation in the presence of spiked levels of levulinic acid, 4-hydroxybenzoic acid, 4-hydroxybenzaldehyde or vanillin

Next, the inhibitory effect of spiking with different concentrations of levulinic acid, 4-hydroxybenzoic acid, 4-hydroxybenzaldehyde or vanillin on the fermentation capacity of GSE16-T18 and MD4 was evaluated in bagasse hydrolysate BH 31B and corn cob hydrolysate CCH 31A. However, no significant and consistent inhibition was observed on fermentation performance of the two yeast strains, even with the highest inhibitor concentrations added (Additional file 1: Fig. S1). We have also evaluated the effect of levulinic acid on the fermentation performance of the GSE16-T18 and MD4 yeast strains in the other hydrolysates: bagasse BH 18, corn cobs CCH 22 and spruce SH 1. The addition of any concentration of levulinic acid did not significantly affect the fermentation performance of the two strains in the three hydrolysates, even in the much more toxic spruce hydrolysate (Additional file 1: Fig. S2).

Inhibitory effects by the phenolic compounds were negligible or non-existing under the tested conditions used in this study. Previously, levulinic acid was described as more inhibitory to glucose fermentation compared to acetic acid or formic acid, while formic acid was identified as more toxic for xylose fermentation compared to levulinic acid, and the latter more inhibitory compared to acetic acid [26]. However, the strong inhibitory effect of levulinic acid is likely due to the high concentration used (4.6 to 18.6 g/L), which is much higher than the concentration generally present in lignocellulose hydrolysates and even higher than the highest concentration ever reported (3.2 g/L) (Table 1). Recently, studies have been published in which the effects of spiking single inhibitors in hydrolysate were evaluated on the fermentation capacity of xylose-fermenting *S. cerevisiae* strain NAPX37 [26, 39]. This strain was evaluated in rape straw hydrolysate containing 3.84 g/L acetic acid, 0.05 g/L formic acid, 0.70 g/L furfural, 0.96 g/L HMF and a total of 0.30 g/L phenolic compounds. The phenolic compounds evaluated (i.e. vanillin, phenol and syringaldehyde) led to the strongest inhibition of glucose and xylose fermentation, followed by the furan aldehydes, whereas addition of weak acids had the smallest inhibitory effect. This is likely due to vanillin being spiked in the hydrolysate in a concentration range of 0.76 to 4.56 g/L, which is again far higher than that reported for lignocellulose hydrolysates [40, 41].

### Evaluation of second-generation yeast fermentation in the presence of spiked levels of formic acid

**Fig. 2:**
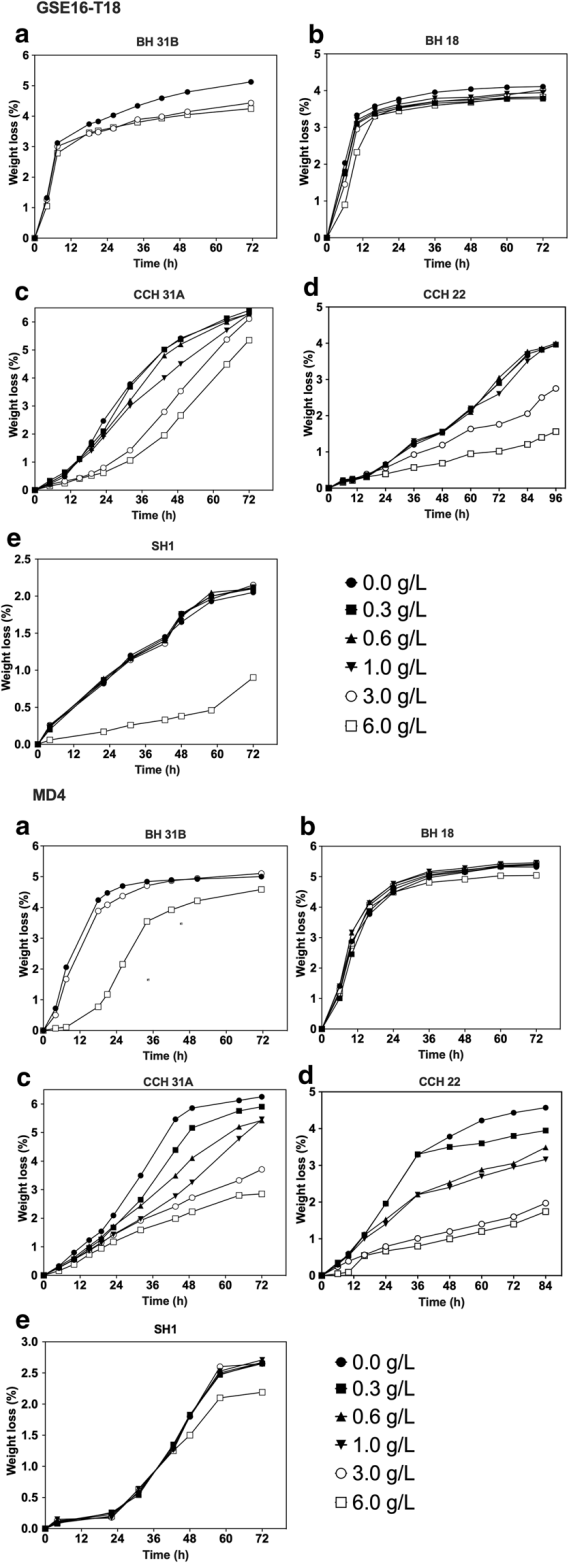
Fermentation performance of GSE16-T18 and MD4 in lignocellulose hydrolysates spiked with formic acid. Small-scale (10 mL) fermentations at pH 5.2, 35 °C, 350 rpm, initial OD_600_ of 5.0. **a** BH 31B, **b** BH 18, **c** CCH 31A, **d** CCH 22 and **e** SH 1, spiked with industrially relevant concentrations of formic acid

The spiking of the bagasse hydrolysates BH 31B and BH18 with different concentrations of formic acid had no or only minor inhibitory effect on the first glucose fermentation phase, except for the highest concentration of 6.0 g/L in BH 31B hydrolysate, but it caused inhibition of the second xylose fermentation phase, and most in BH 31B by strain GSE16-T18 (Fig. 2). Xylose fermentation by strain MD4 appeared to be somewhat more tolerant to formic acid although its glucose fermentation was more affected (Fig. 2). Moreover, equimolar concentrations of formic acid appeared slightly more inhibitory to BH 31B compared to BH 18. This might be explained by differences in the synergistic effects between the inhibitors present, due to the somewhat divergent inhibitor composition of the two hydrolysates. The fermentation of the two corn cob hydrolysates CCH 31A and CCH 22 was much slower than that of the bagasse hydrolysates and in this case, spiking with formic acid caused a clear, concentration dependent additional inhibition of the fermentation rate (Fig. 2). This suggests that formic acid acts as a major inhibitor in the two corn cob hydrolysates and may be responsible at least in part for their slower fermentation. On the other hand, the slow fermentation of spruce hydrolysate was further reduced only in the presence of the highest concentration of spiked formic acid (Fig. 2), suggesting that formic acid is not a major culprit for the slow fermentation of this spruce hydrolysate. The final ethanol titer at the end of the fermentation (72 h) was also measured (Additional file 1: Table S1). The results were consistent with the reduction in weight loss at the end of the fermentation compared to the weight loss of the unspiked hydrolysate (Fig. 2). Whereas MD4 generally showed a better fermentation performance in unspiked lignocellulose hydrolysates compared to GSE16-T18, MD4 turned out to be more sensitive to spiked formic acid in most hydrolysates compared to GSE16-T18 (Fig. 2).

The inhibitory threshold concentration of formic acid that affected fermentation capacity of GSE16-T18 and MD4 was highly variable depending on the hydrolysate. This weak acid exerted inhibitory effects in certain hydrolysates in presence of only 0.3 g/L, but most of the toxic effects were observed in presence of at least 3.0 g/L formic acid. Although most lignocellulose hydrolysates contain less than 3 g/L formic acid, hydrolysate from hardwood chips and steam explosion pre-treated corn stover were found to contain, respectively, 4.6 and 6.8 g/L formic acid (Table 1).

### Evaluation of second-generation yeast fermentation in the presence of spiked levels of acetic acid

**Fig. 3:**
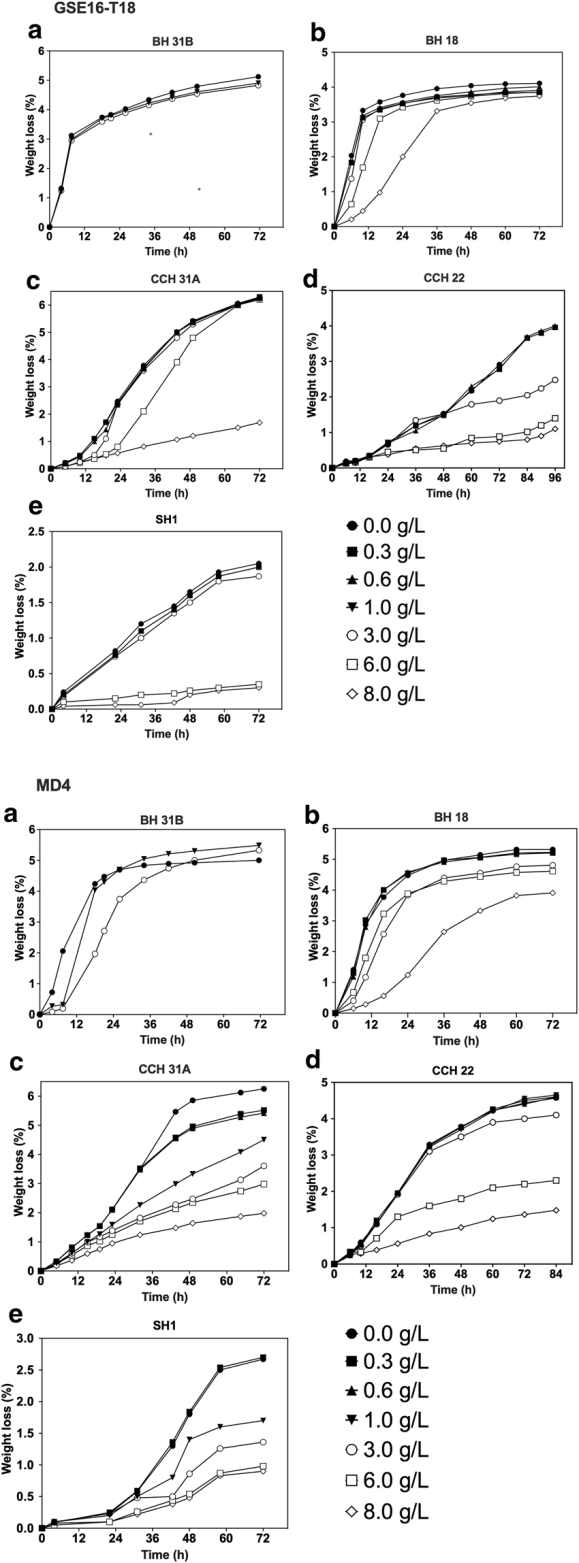
Fermentation performance of GSE16-T18 and MD4 in lignocellulose hydrolysates spiked with acetic acid. Small-scale (10 mL) fermentations at pH 5.2, 35 °C, 350 rpm, initial OD_600_ of 5.0. **a** BH 31B, **b** BH 18, **c** CCH 31A, **d** CCH 22 and **e** SH 1, spiked with industrially relevant concentrations of acetic acid

The spiking of the lignocellulose hydrolysates with acetic acid in general caused a similar inhibition as with formic acid (Figs. 2, 3). In the BH 18 and SH1 hydrolysates, acetic acid was somewhat more inhibitory than formic acid, while in the BH 31B and CCH 31A hydrolysates, it was the reverse, but there was also some variation between the two strains (Figs. 2, 3). The different levels of inhibition with BH 31B and BH 18 in hydrolysates from the same origin, might be explained by the somewhat divergent inhibitor composition that could lead to different synergistic effects between the multiple inhibitors present. The main differences between the hydrolysates and the two strains were in general also comparable for formic and acetic acid. This suggests that acetic acid may also serve as a major inhibitor in corn cob hydrolysate (Fig. 3). The strongest difference with formic acid was observed with the MD4 strain in spruce hydrolysate where acetic acid caused considerable inhibition, suggesting that it may also be a major inhibitor in this type of hydrolysate. The ethanol titer measured at the end of the fermentation (72 h) correlated well with the differences in weight loss between the unspiked and spiked hydrolysates measured after the same time period (Additional file 1: Table S2). As observed for formic acid, MD4 was also more sensitive to spiking with acetic acid compared to GSE16-T18 (Fig. 3).

In other studies, formic acid was the most toxic of the two weak acids on fermentation capacity of *S. cerevisiae* in synthetic medium, at equimolar concentrations [21, 42]. This is likely due to the more toxic nature of the anion form of formic acid [21]. Notwithstanding the fact that acetic acid is most often the inhibitor present at highest concentration in lignocellulose hydrolysates (usually in a range of 4.0 to 6.0 g/L), both yeast strains appeared only inhibited in presence of at least 6.3 g/L spiked acetic acid. Nevertheless, cheaper pre-treatment methods, such as acid- or alkali-based hydrolysis, generally result in higher concentrations of acetic acid (up to 11.0 g/L) [15, 18, 19]. In addition, tolerance to weak acids is often linked to tolerance to low pH, and during 2G fermentations pH drops below 5, which strongly affects cell viability [43]. Improving weak acid tolerance not only holds great promise to improve fermentation capacity, but also to reduce bacterial contamination under process conditions with a pH below 5 [44].

### Evaluation of second-generation yeast fermentation in the presence of spiked levels of 5-hydroxymethylfurfural

**Fig. 4:**
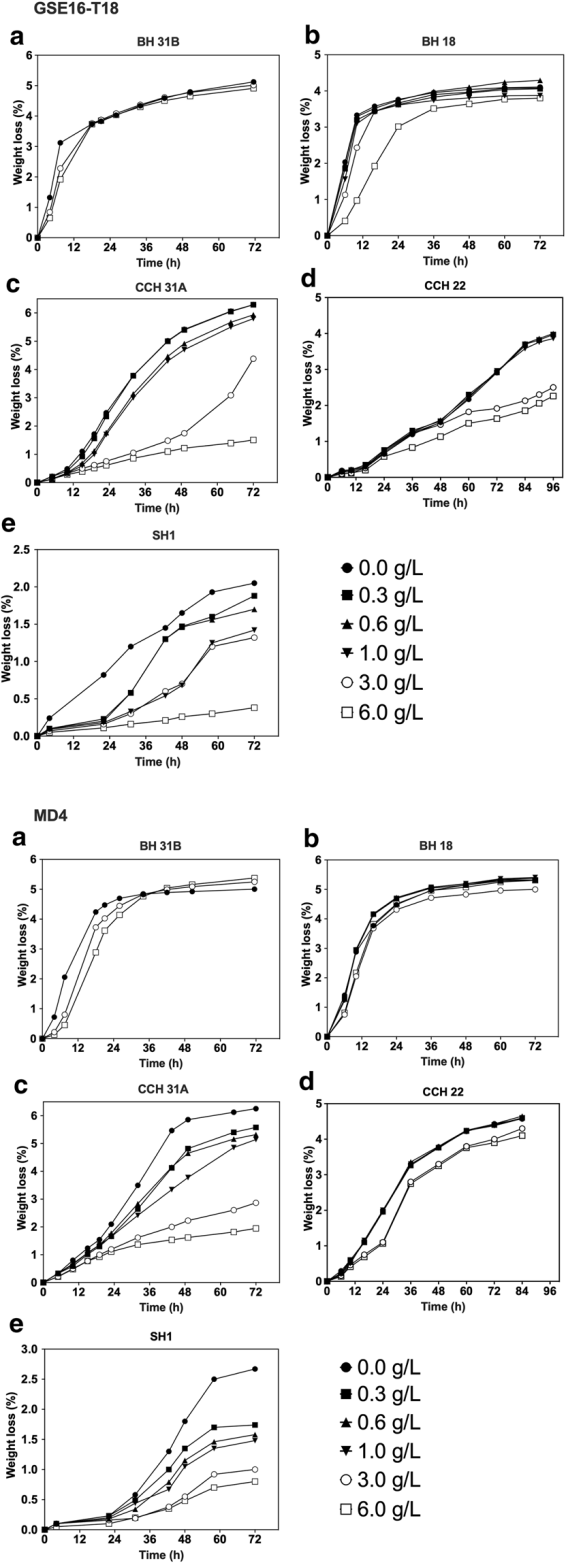
Fermentation performance of GSE16-T18 and MD4 in lignocellulose hydrolysates spiked with HMF. Small-scale (10 mL) fermentations at pH 5.2, 35 °C, 350 rpm, initial OD_600_ of 5.0. **a** BH 31B, **b** BH 18, **c** CCH 31A, **d** CCH 22 and **e** SH 1 spiked with industrially relevant concentrations of HMF

Next, the fermentation performance in the same hydrolysates spiked with HMF was evaluated (Fig. 4). Compared to formic and acetic acid, HMF was much more inhibitory at equivalent concentrations. As observed with formic and acetic acid, addition of HMF in the bagasse hydrolysates caused least inhibition. In the corn cob hydrolysates, HMF caused significant inhibition and in the spruce hydrolysate, HMF inhibition was even more pronounced, with a strong effect already at the lowest concentrations (Fig. 4). The MD4 strain nearly always performed somewhat better compared to GSE16-T18. The results suggest that in corn cob hydrolysate and spruce hydrolysate, HMF acts as a major inhibitor and therefore, even a slight increase in concentration, caused by the spiked HMF, causes a significant or even a drastic inhibition of the fermentation. The measurements of the final ethanol titer after 72 h of fermentation generally agreed with the results from the weight loss determinations (Additional file 1: Table S3). As opposed to the higher sensitivity of MD4 to formic and acetic acid compared to GSE16-T18, this difference was either smaller or absent for inhibition with spiked HMF (Fig. 4).

### Evaluation of second-generation yeast fermentation in the presence of spiked levels of furfural

**Fig. 5:**
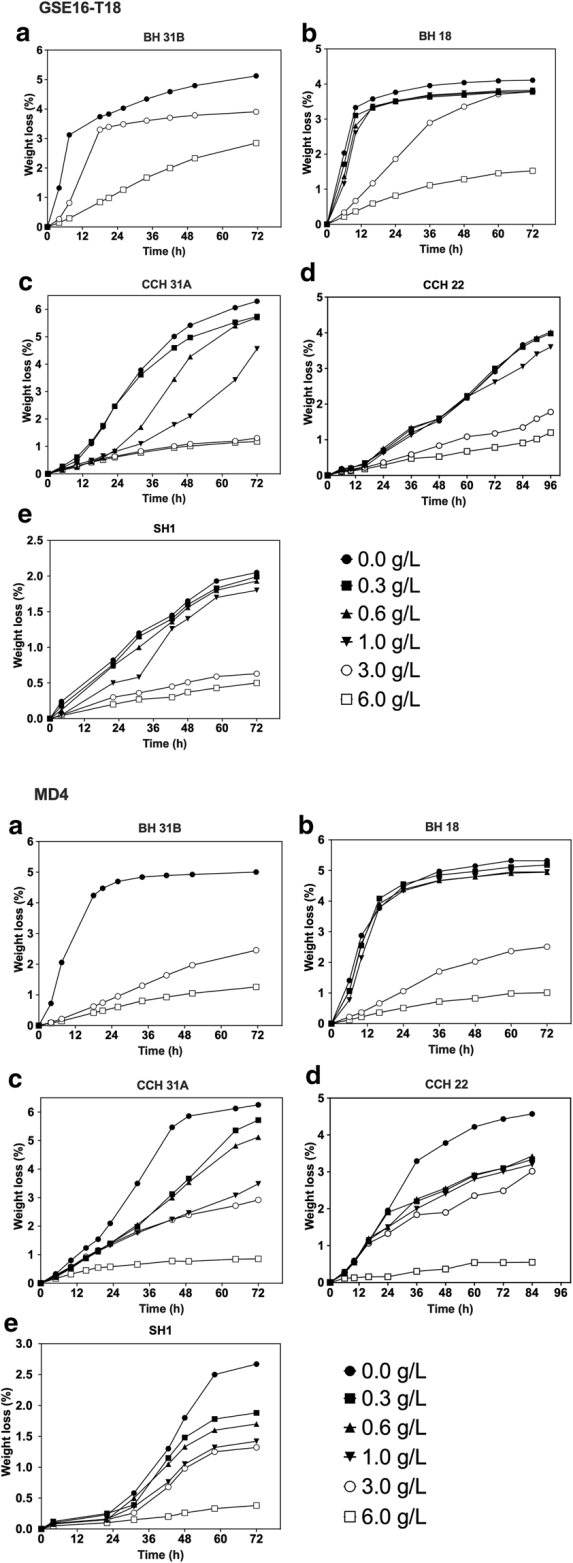
Fermentation performance of GSE16-T18 and MD4 in lignocellulose hydrolysates spiked with furfural. Small-scale (10 mL) fermentations at pH 5.2, 35 °C, 350 rpm, initial OD_600_ of 5.0. **a** BH 31B, **b** BH 18, **c** CCH 31A, **d** CCH 22 and **e** SH 1 spiked with industrially relevant concentrations of furfural

**Fig. 6:**
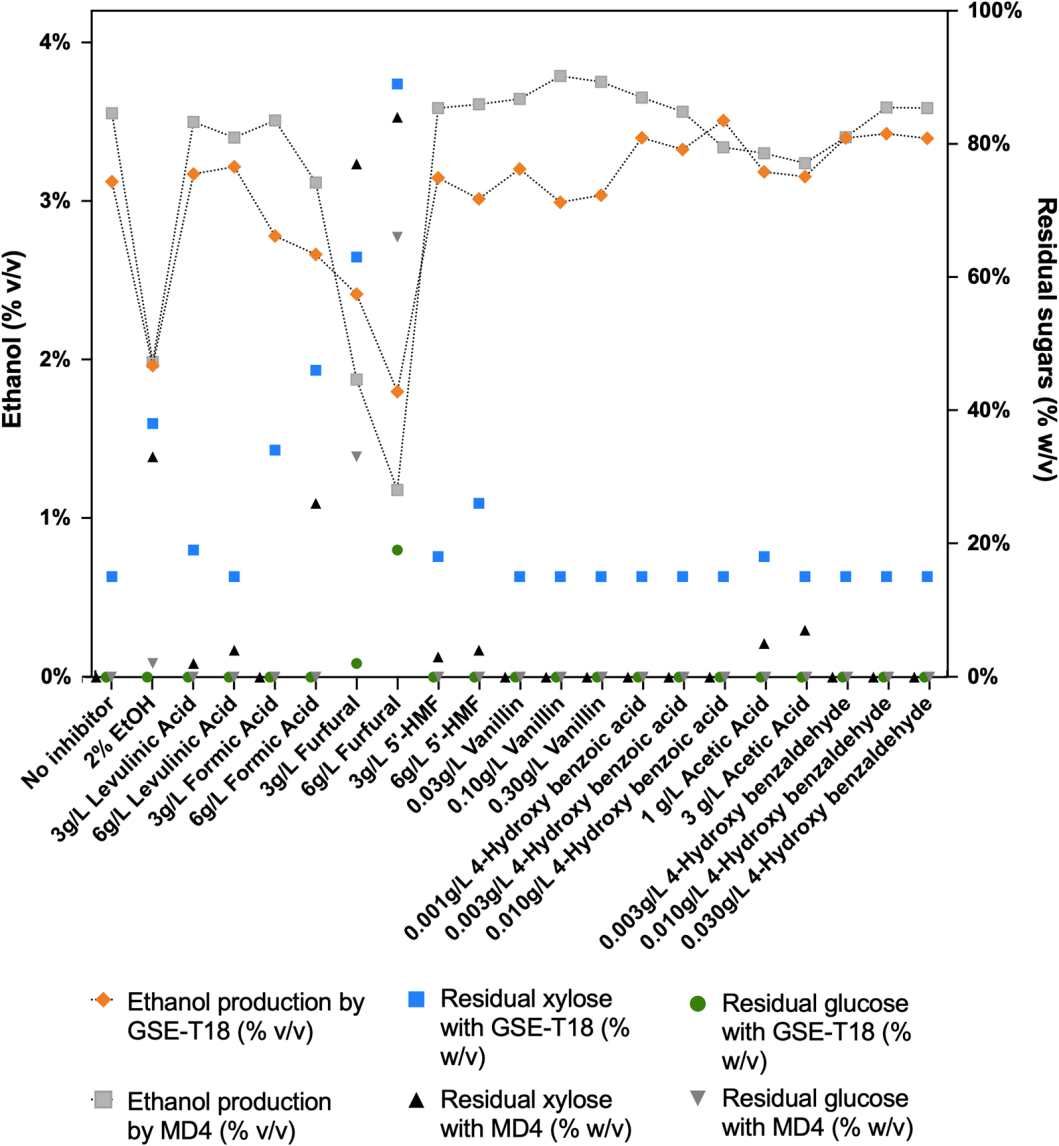
Ethanol titer and residual sugar content after fermentation by 2G yeast strains GSE16-T18 and MD4 in bagasse hydrolysate BH 31B spiked with different inhibitors. Overview of ethanol titer, and residual xylose and glucose concentrations determined with HPLC in small-scale fermentations with BH 31B, spiked with specific concentrations of fermentation inhibitors. Percentage ethanol produced after 72 h (left axis) and percentage residual glucose and xylose (right axis) are shown. In the fermentation where 2% ethanol was added as inhibitor, this addition was subtracted from the final ethanol titer reached after 72 h

Subsequently, the performance of the GSE16-T18 and MD4 strains in the same hydrolysates spiked with furfural was evaluated (Fig. 5). It turned out to be the most toxic of all inhibitors tested. Even in the bagasse hydrolysates, there was strong inhibition in concentrations that caused only very limited inhibition with HMF or with formic and acetic acid. This indicates that furfural is likely the major inhibitor in bagasse hydrolysates. Also in the corn and spruce hydrolysates, furfural caused stronger inhibition than HMF (Fig. 4, 5). Furfural is present in concentrations of up to 3.5 g/L in most lignocellulose hydrolysates or even higher for some biomass types depending on the pre-treatment (Table 1). Addition of concentrations as low as 0.3 g/L furfural already reduced the xylose fermentation phase in certain hydrolysates. Hence, furfural is likely the major inhibitor in all lignocellulose hydrolysates investigated. The final ethanol titer determined after 72 h of fermentation again correlated well with the weight loss measurements (Additional file 1: Table S4). HPLC analysis revealed that furfural was the only inhibitor that also resulted in residual glucose levels with both yeast strains in BH 31B bagasse hydrolysate (Fig. 6, Table 3). The MD4 strain was more sensitive to spiked furfural in all lignocellulose hydrolysates compared to the GSE16-T18 strain (Figs. 5, 6).

**Table 3:** Overview of ethanol yield, ethanol productivity, percentage of the theoretical maximum yield and sugar conversion by GSE16-T18 and MD4 in bagasse hydrolysate BH 31B spiked with different inhibitors

	Ethanol yield (g/g) by GSE16-T18	Ethanol yield (g/g) by MD4	Ethanol productivity (g/L/h) by GSE16-T18	Ethanol productivity (g/L/h) by MD4	% of theoretical maximum yield by GSE16-T18	% of theoretical maximum yield by MD4	Glucose conversion (%) by GSE16-T18	Xylose conversion (%) by GSE16-T18	Glucose conversion (%) by MD4	Xylose conversion (%) by MD4
No inhibitor	0.30	0.34	0.41	0.47	58	66	99	84	99	99
2% EtOH	0.19	0.19	0.26	0.26	37	37	99	59	98	64
3 g/L Levulinic Acid	0.30	0.33	0.42	0.46	59	65	99	80	99	98
6 g/L Levulinic Acid	0.31	0.32	0.43	0.45	60	63	99	85	99	95
3 g/L Formic Acid	0.26	0.33	0.37	0.46	52	66	99	63	99	99
6 g/L Formic Acid	0.25	0.30	0.35	0.41	50	58	99	50	99	72
3 g/L Furfural	0.23	0.18	0.32	0.25	45	35	98	32	54	17
6 g/L Furfural	0.17	0.11	0.24	0.16	34	22	73	4	7	9
3 g/L 5′-HMF	0.30	0.34	0.42	0.47	59	67	99	81	99	96
6 g/L 5′-HMF	0.29	0.34	0.40	0.48	56	67	99	72	99	96
0.03 g/L Vanillin	0.30	0.35	0.42	0.48	60	68	99	89	99	99
0.10 g/L Vanillin	0.28	0.36	0.40	0.50	56	71	99	89	99	99
0.30 g/L Vanillin	0.29	0.36	0.40	0.50	57	70	99	93	99	98
0.001 g/L 4-Hydroxy benzoic acid	0.32	0.35	0.45	0.48	63	68	99	95	99	99
0.003 g/L 4-Hydroxy benzoic acid	0.32	0.34	0.44	0.47	62	67	99	93	99	99
0.010 g/L 4-Hydroxy benzoic acid	0.33	0.32	0.46	0.44	65	62	99	93	99	99
0.1% Acetic Acid	0.30	0.31	0.42	0.44	59	62	99	80	99	95
0.3% Acetic Acid	0.30	0.31	0.42	0.43	59	60	99	88	99	93
0.003 g/L 4-Hydroxy benzaldehyde	0.32	0.32	0.45	0.45	63	64	99	90	99	99
0.010 g/L 4-Hydroxy benzaldehyde	0.33	0.34	0.45	0.47	64	67	99	94	99	99
0.030 g/L 4-Hydroxy benzaldehyde	0.32	0.34	0.45	0.47	63	67	99	95	99	99

In the fermentation where 2% ethanol was added as inhibitor, this addition was subtracted from the final ethanol yield reached after 72 h

A previous study evaluated the effect of different inhibitors on the fermentation performance of strain NAPX37 [26]. In this research, HMF was added in a range from 0.63 to 3.78 g/L and furfural from 0.48 to 2.88 g/L. Even compared to the much higher weak acid levels spiked in this research, both HMF and furfural appeared more inhibitory. HMF and furfural clearly affected the lag phase of strain NAPX37, but fermentation of glucose appeared complete after 48 h. In contrast, in this study, the presence of industrially relevant concentrations of furfural resulted in residual glucose and xylose after 72 h. However, both GSE16-T18 and MD4 have a similar fermentation profile in non-spiked bagasse hydrolysate compared to NAPX37 in rape straw hydrolysate, completing both xylose and glucose fermentation in 48 h. Possibly, NAPX37 is more inhibitor tolerant compared to the yeast strains used in this study, or the rape straw hydrolysate might be less toxic compared to the lignocellulose hydrolysates used in our study. Prior to being engineered for xylose fermentation, KF-7, the parent strain of NAPX37, was described as an efficient 2G bioethanol producer in different hydrolysates [45, 46].

Presence of the furan aldehydes leads to the formation of reactive oxygen species (ROS) in the cells, increases sensitivity to osmotic and salt stress, and inhibits carbon metabolism by inhibition of glycolytic enzymes [47]. A major cause of furan aldehyde inhibition was proposed to be drainage of redox power [48]. In addition, it has been reported that in the simultaneous presence of both furan aldehydes, furfural appears to be converted first into lesser toxic compounds, before the onset of HMF conversion [21, 38]. This may be linked to the higher hydrophobicity of furfural because it lacks the hydroxymethyl group present in HMF. The detrimental effects exerted by HMF on the yeast cells may thus be longer lasting than those of furfural.

### Overview of the effect of fermentation inhibitors on ethanol titer and residual sugar levels

In Fig. 6, the effect of different concentrations of all inhibitors investigated on the ethanol titer and residual levels of glucose and xylose is shown after 72 h of fermentation in BH 31B bagasse hydrolysate with the two yeast strains GSE16-T18 and MD4. The corresponding data for ethanol yield, ethanol productivity, percentage of the theoretical maximum yield and sugar conversion are shown in Table 3. The data again show that furfural is the most toxic inhibitor and also the only inhibitor that leaves high levels of residual glucose. Significant levels of residual xylose are also observed with formic and acetic acid. The effect of 2% ethanol addition was also tested. It caused a strong reduction in the final ethanol titer, yield and productivity with both yeast strains and also caused high residual levels of xylose, while glucose was completely fermented (Fig. 6, Table 3). A concentration of 2% ethanol has no toxicity for yeast and the total level present at the end of the fermentation was only 4%. This indicates that the ethanol produced in the 2G bioethanol production from the lignocellulose hydrolysates may serve as a major inhibitor by increasing the toxicity of the other inhibitory compounds. Ethanol is well known to enhance the toxicity of many stress factors and may increase the toxicity of chemical compounds by enhancing membrane permeability [49–52].

In addition to the negative effect of the spiked inhibitors on ethanol titer, yield and productivity, an extension of the lag phase, with limited effects on the other parameters, was also observed with some compounds in particular hydrolysates, such as for fermentation by GSE16-T18, in the presence of spiked formic acid or acetic acid in CCH 31A hydrolysate (Figs. 2, 3). This is likely due not only to the spiked inhibitor but to synergistic effects with the other inhibitory compounds in the hydrolysate [11].

## Conclusion

The relevance of different inhibitors in five lignocellulose hydrolysates was determined by spiking with industrially relevant concentrations. Spiked furfural was most toxic followed by HMF, acetic acid and formic acid, while spiking with levulinic acid, 4-hydroxybenzaldehyde, 4-hydroxybenzoic acid or vanillin caused little inhibition compared to unspiked hydrolysate. Our results highlight the importance of individual inhibitor evaluation in a medium containing a genuine mix of inhibitors as well as the ethanol produced during the fermentation. The further evolved MD4 strain generally showed superior performance compared to GSE16-T18, highlighting the potential of increasing yeast inhibitor tolerance for improving the industrial process economics.

## Materials and methods

### Yeast strains and cultivation media

The yeast strains used in this work were GSE16-T18 and MD4. MCB collection numbers of these strains are JT 24753 and JT 28503, respectively. GSE16-T18 was obtained by evolutionary engineering from strain GS1.11–26 [37]. MD4 is a 2G bioethanol strain obtained by repeated backcrossing of strain GSE16-T18 with strain HDY.GUF5 [37].

### Small-scale fermentations in lignocellulose hydrolysates

Inhibitor tolerance of T18 and MD4 was evaluated in lignocellulose hydrolysates from corn cob, bagasse and spruce (composition depicted in Table 2) that were spiked with a range of industrially relevant inhibitor concentrations. After pre-culture of the yeast strains for 48 h at 30 °C with shaking at 200 rpm in YPD2% (10 g/L yeast extract, 20 g/L bacteriological peptone, 2% D-glucose) up to stationary phase, small-scale (10 mL) semi-anaerobic fermentations with the 2G yeast strains MD4 and T18 were performed at pH 5.2, 35 °C, magnetic stirring at 350 rpm, and a yeast inoculum OD 5.0. Weight loss of the fermentation tubes, which is correlated with CO_2_ production during conversion of glucose and xylose into ethanol, was measured continuously, and sampling at different time points was performed to analyze sugar and inhibitor concentrations by HPLC (Model 10AD Shimadzu chromatograph via refractive index and UV–visible detection), after filtering the hydrolysate samples. Ethanol, glucose and xylose concentrations were determined using the Bio-Rad carbohydrate analysis column Aminex HPX 87H 300X7 8 mm column, at 45 °C, with 5 mM sulfuric acid as eluent at 0.6 mL/min.

In Table 3, ethanol yield (g/g) was calculated as the ethanol titer (w/v) obtained divided by the total amount of D-glucose and D-xylose present in the hydrolysate. Percentage of the theoretical maximum yield is calculated as the fraction of the ethanol yield divided by the theoretical maximum yield. Maximum ethanol yield was obtained as maximal ethanol titer theoretically obtained from D-glucose and D-xylose levels present in the hydrolysate divided by the total amount of D-glucose and D-xylose present in the hydrolysate. Ethanol productivity (g/L/h) was calculated as maximum ethanol yield multiplied by percentage of the theoretical maximum yield, in 1 L hydrolysate and 1 h. Glucose and xylose conversions (%) were determined as the fraction of D-glucose or D-xylose that was converted divided by the total initial D-glucose or D-xylose levels present in the hydrolysate.

### Reproducibility of the results

All experiments were repeated at least once using several inhibitor concentrations and two different yeast strains. Representative results are shown.

## Supplementary Information

**Additional file 1.** Additional figures and tables.

## Data Availability

All data have been stored on dedicated computers at KU Leuven. All data and yeast strains are freely available upon request.

## Abbreviations

2G: Second-generation
HMF: Hydroxymethylfurfural
