# Supplementary material for: Identification of the major fermentation inhibitors of recombinant 2G yeasts in diverse lignocellulose hydrolysates
Source: Biotechnol Biofuels. 2021 Apr 9;14:92. doi: 10.1186/s13068-021-01935-9 (PMC8034183; doi:10.1186/s13068-021-01935-9)
Supplement: Supplementary file 1 — Additional file 1. Additional figures and tables. [file 13068_2021_1935_MOESM1_ESM.docx]

**Supplementary Information**

**Supplementary Figures**

**
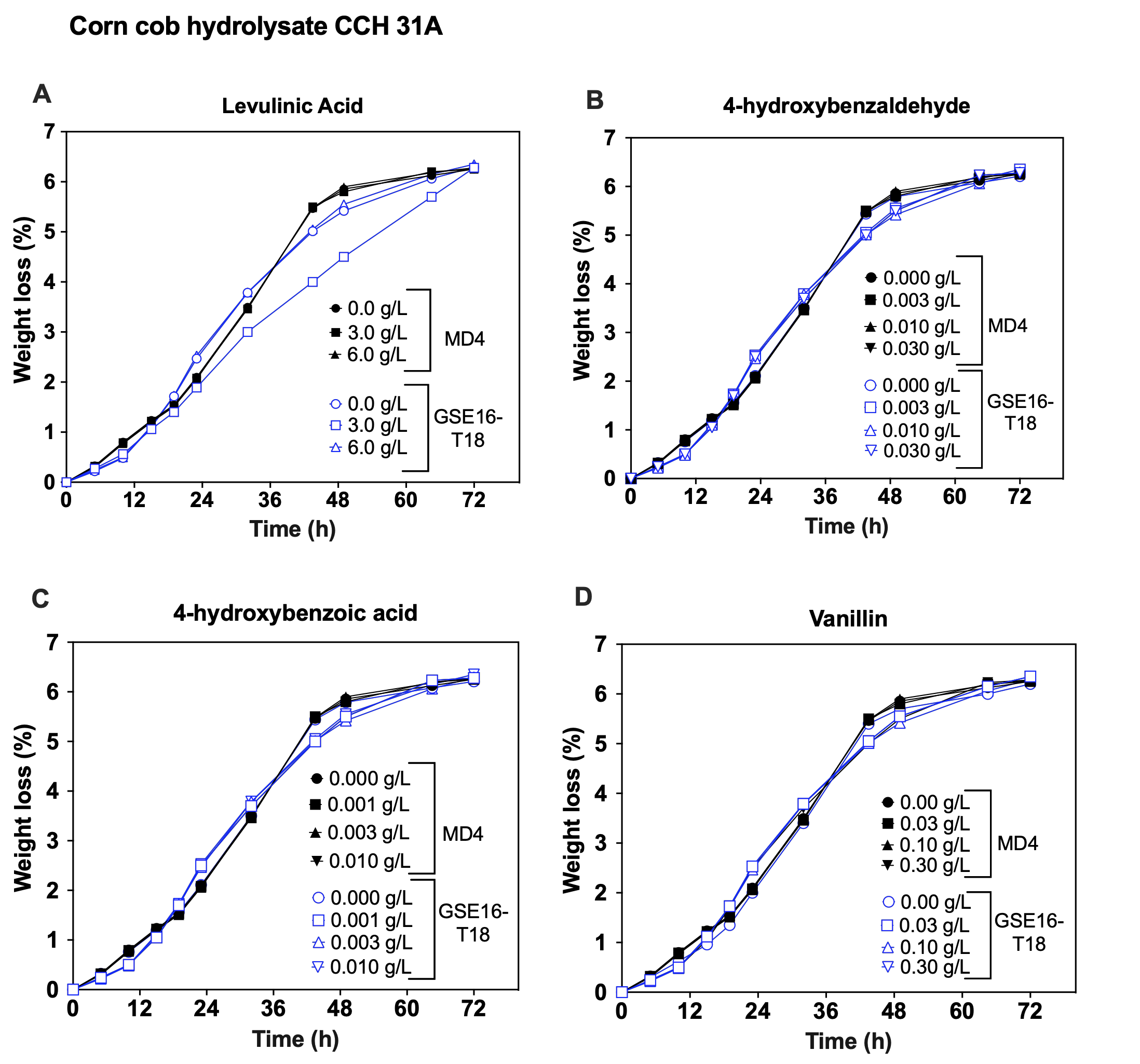

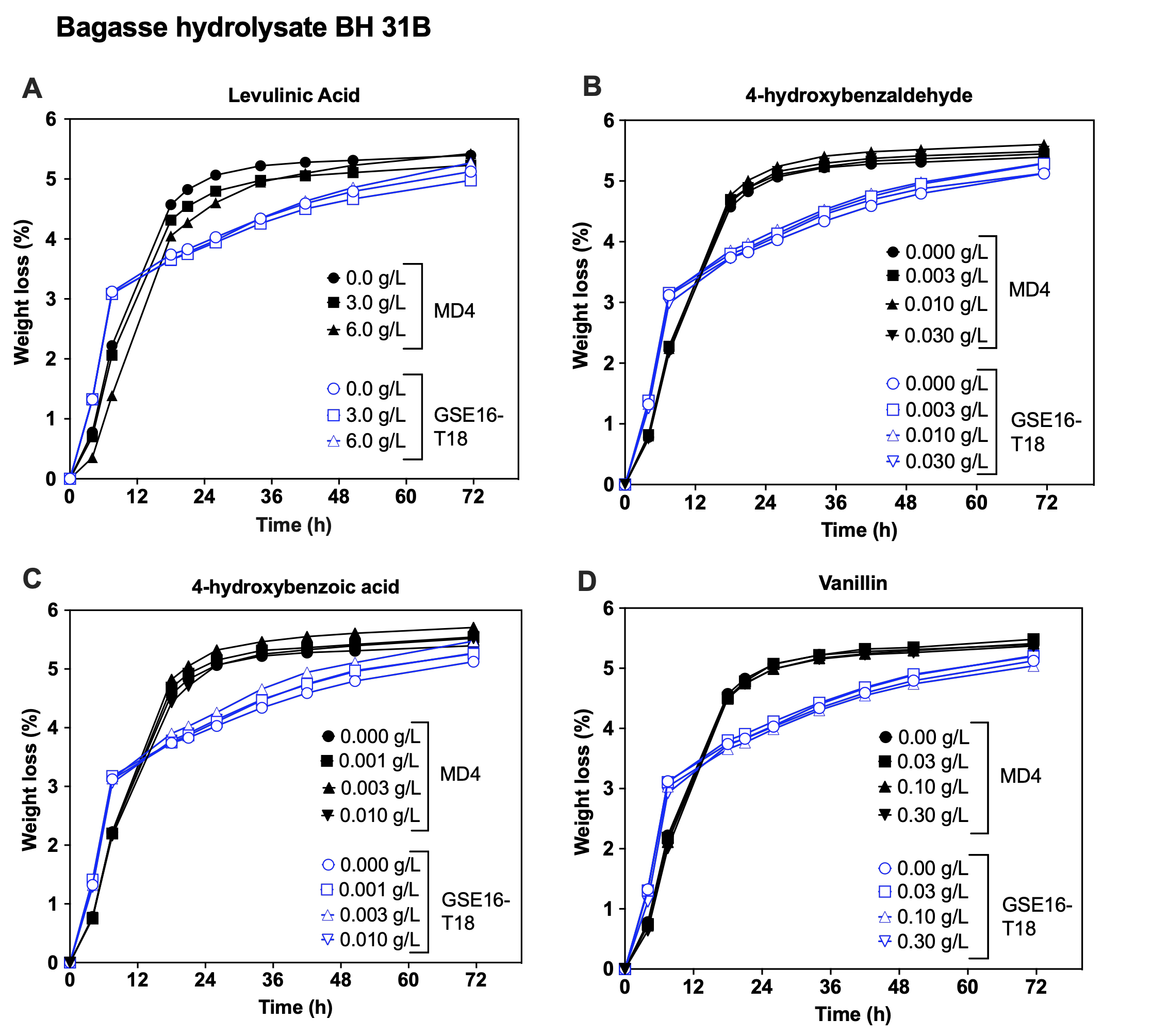
**

**Supplementary Figure 1. Fermentation performance of 2G yeast strains in lignocellulose hydrolysates with spiked inhibitors.** (Upper part) Bagasse hydrolysate BH 31B, (Lower part) Corn cob hydrolysate CCH 31A. Small-scale (10 mL) fermentations with MD4 or GSE16-T18, pH 5.2, 35°C, 350 rpm, initial OD_600_ of 5.0, spiked with different industrially relevant concentrations of (A) levulinic acid, (B) 4-hydroxybenzaldehyde, (C) 4-hydroxybenzoic acid and (D) vanillin.


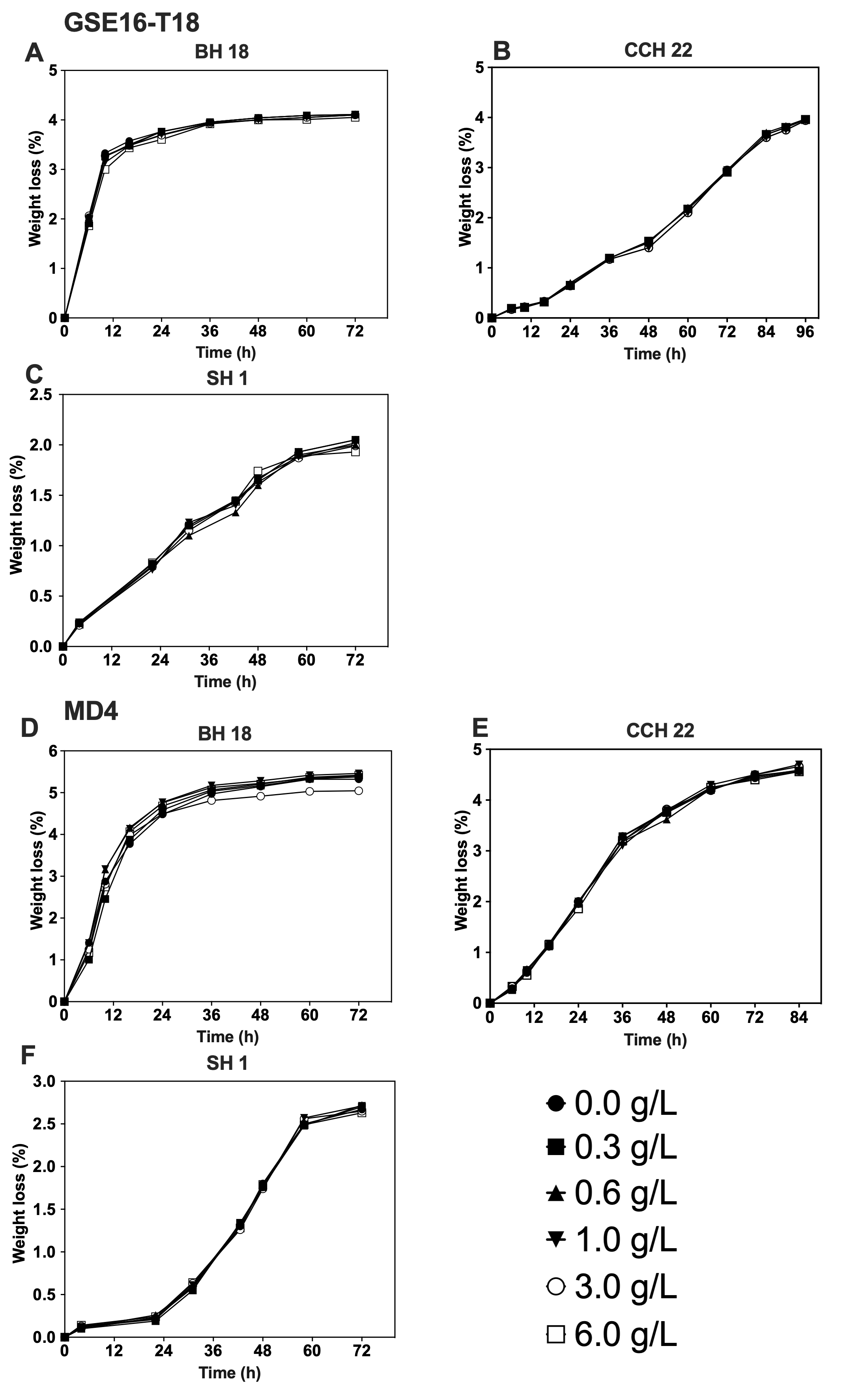


**Supplementary Figure 2. Fermentation performance of 2G yeast strains in lignocellulose hydrolysates spiked with levulinic acid.** Small-scale (10 mL) fermentations with MD4 (A-C) and GSE16-T18 (D-F) at pH 5.2, 35°C, 350 rpm, initial OD_600_ of 5.0. (A, D) Bagasse BH 18, (B, E) Corn cobs CCH 22, (C, F) Spruce SH 1 spiked with industrially relevant concentrations of levulinic acid.

**Supplementary Tables**

**Supplementary Table 1. Effect of formic acid on final ethanol titer obtained with GSE16-T18 and MD4.** Small-scale (10 mL) fermentations were performed with GSE16-T18 and MD4 in hydrolysates spiked with formic acid. The final ethanol titer (%, w/v) after 72 h, as measured by HPLC, is indicated. The ethanol titer is also shown as a percentage of that in unspiked hydrolysate. –, this condition was not evaluated.

| **Condition** | **BH 31B**  **(%, w/v)** | **BH 18**  **(%, w/v)** | **CCH 31A**  **(%, w/v)** | **CCH 22**  **(%, w/v)** | **SH 1**  **(%, w/v)** |
| --- | --- | --- | --- | --- | --- |
| **Ethanol titer after 72 h fermentation with GSE16-T18** | | | | | |
| **Unspiked hydrolysate** | 5.12  (100 %) | 4.11  (100 %) | 6.25  (100 %) | 3.96  (100 %) | 2.05  (100 %) |
| **0.6 g/L formic acid** | - | 4.11  (100 %) | 6.26  (100 %) | 3.94  (99 %) | 2.02  (99 %) |
| **1.0 g/L formic acid** | - | 4.05  (98 %) | 6.24  (100 %) | 3.94  (99 %) | 2.06  (100 %) |
| **3.0 g/L formic acid** | 4.44  (87 %) | 3.94  (95 %) | 6.11  (98 %) | 2.75  (69 %) | 2.03  (99 %) |
| **6.0 g/L formic acid** | 4.25  (83 %) | 3.82  (93 %) | 5.35  (86 %) | 1.56  (39 %) | 0.91  (44 %) |
| **Ethanol titer after 72 h fermentation with MD4** | | | | | |
| **Unspiked hydrolysate** | 5.05  (100 %) | 5.32  (100 %) | 6.29  (100 %) | 4.57  (100 %) | 2.67  (100 %) |
| **0.6 g/L formic acid** | - | 5.30  (99 %) | 5.46  (87 %) | 3.49  (76 %) | 2.65  (99 %) |
| **1.0 g/L formic acid** | - | 5.28  (99 %) | 5.42  (86 %) | 3.16  (69 %) | 2.67  (100 %) |
| **3.0 g/L formic acid** | 5.11  (101 %) | 5.31  (99 %) | 3.71  (59 %) | 1.97  (43 %) | 2.67  (100 %) |
| **6.0 g/L formic acid** | 4.54  (89 %) | 5.04  (95 %) | 2.85  (45 %) | 1.74  (38 %) | 2.19  (82 %) |

**Supplementary Table 2. Effect of acetic acid on final ethanol titer obtained with GSE16-T18 and MD4.** Small-scale (10 mL) fermentations were performed with GSE16-T18 and MD4 in hydrolysates spiked with acetic acid. The final ethanol titer (%, w/v) after 72 h, as measured by HPLC, is indicated. The ethanol titer is also shown as a percentage of that in unspiked hydrolysate. –, indicates that this condition was not evaluated.

| **Condition** | **BH 31B**  **(%, w/v)** | **BH 18**  **(%, w/v)** | **CCH 31A**  **(%, w/v)** | **CCH 22**  **(%, w/v)** | **SH 1**  **(%, w/v)** |
| --- | --- | --- | --- | --- | --- |
| **Ethanol titer after 72 h fermentation with GSE16-T18** | | | | | |
| **Unspiked hydrolysate** | 5.12  (100 %) | 4.11  (100 %) | 6.25  (100 %) | 3.96  (100 %) | 2.05  (100 %) |
| **0.6 g/L acetic acid** | - | 4.00  (97 %) | 6.29  (101 %) | 3.97  (100 %) | 2.00  (98 %) |
| **1.0 g/L acetic acid** | 4.91  (96 %) | 4.00  (97 %) | 6.28  (101 %) | 3.95  (100 %) | 2.04  (99 %) |
| **3.0 g/L acetic acid** | 4.82  (94 %) | 3.90  (95 %) | 6.25  (100 %) | 2.48  (63 %) | 1.87  (91 %) |
| **6.0 g/L acetic acid** | - | 3.85  (94 %) | 6.20  (99 %) | 2.20  (56 %) | 0.35  (17 %) |
| **8.0 g/L acetic acid** | - | 3.74  (91%) | 1.69  (27 %) | 2.10  (53 %) | 0.30  (15 %) |
| **Ethanol titer after 72 h fermentation with MD4** | | | | | |
| **Unspiked hydrolysate** | 5.05  (100 %) | 5.32  (100 %) | 6.29  (100 %) | 4.57  (100 %) | 2.67  (100 %) |
| **0.6 g/L acetic acid** | - | 5.28  (99 %) | 5.42  (86 %) | 3.48  (76 %) | 2.67  (100 %) |
| **1.0 g/L acetic acid** | 5.49  (109 %) | 5.22  (98 %) | 4.51  (72 %) | 3.16  (69 %) | 1.70  (64 %) |
| **3.0 g/L acetic acid** | 5.32  (105 %) | 4.62  (87 %) | 3.60  (57 %) | 1.97  (43 %) | 1.36  (51 %) |
| **6.0 g/L acetic acid** | - | 4.55  (86 %) | 2.98  (47 %) | 1.74  (38 %) | 0.98  (37 %) |
| **8.0 g/L acetic acid** | - | 3.93  (74 %) | 1.98  (31 %) | 0.80  (18 %) | 0.90  (34 %) |

**Supplementary Table 3. Effect of HMF on final ethanol titer obtained with GSE16-T18 and MD4.** Small-scale (10 mL) fermentations were performed with GSE16-T18 and MD4 in hydrolysates spiked with HMF. The final ethanol titer (%, w/v) after 72 h, as measured by HPLC, is indicated. The ethanol titer is also shown as a percentage of that in unspiked hydrolysate. – indicates that this condition was not evaluated.

| **Condition** | **BH 31B**  **(%, w/v)** | **BH 18**  **(%, w/v)** | **CCH 31A**  **(%, w/v)** | **CCH 22**  **(%, w/v)** | **SH 1**  **(%, w/v)** |
| --- | --- | --- | --- | --- | --- |
| **Ethanol titer after 72 h fermentation with GSE16-T18** | | | | | |
| **Unspiked hydrolysate** | 5.12  (100 %) | 4.11  (100 %) | 6.25  (100 %) | 3.96  (100 %) | 2.05  (100 %) |
| **0.6 g/L HMF** | - | 4.29  (104 %) | 5.93  (95 %) | 3.93  (99 %) | 1.70  (83 %) |
| **1.0 g/L HMF** | - | 3.87  (94 %) | 5.80  (93 %) | 3.86  (97 %) | 1.42  (69 %) |
| **3.0 g/L HMF** | 5.02  (98 %) | 4.10  (100 %) | 4.38  (70 %) | 2.50  (63 %) | 1.32  (64 %) |
| **6.0 g/L HMF** | 4.91  (96 %) | 3.80  (92 %) | 1.50  (24 %) | 2.26  (57 %) | 0.38  (19 %) |
| **Ethanol titer after 72 h fermentation with MD4** | | | | | |
| **Unspiked hydrolysate** | 5.05  (100 %) | 5.32  (100 %) | 6.29  (100 %) | 4.57  (100 %) | 2.67  (100 %) |
| **0.6 g/L HMF** | - | 5.37  (101 %) | 5.32  (85 %) | 4.57  (100 %) | 1.58  (59 %) |
| **1.0 g/L HMF** | - | 5.32  (100 %) | 5.14  (82 %) | 4.58  (100 %) | 1.48  (55 %) |
| **3.0 g/L HMF** | 5.37  (106 %) | 5.30  (99 %) | 2.86  (45 %) | 4.30  (94 %) | 1.00  (37 %) |
| **6.0 g/L HMF** | 5.24  (104 %) | 5.00  (94 %) | 1.95  (31 %) | 4.10  (90 %) | 0.80  (30 %) |

**Supplementary Table 4. Effect of furfural on final ethanol titer obtained with GSE16-T18 and MD4.** Small-scale (10 mL) fermentations were performed with GSE16-T18 and MD4 in hydrolysates spiked with furfural. The final ethanol titer (%, w/v) after 72 h, as measured by HPLC, is indicated. The ethanol titer is also shown as a percentage of that in unspiked hydrolysate. – indicates that this condition was not evaluated.

| **Condition** | **BH 31B**  **(%, w/v)** | **BH 18**  **(%, w/v)** | **CCH 31A**  **(%, w/v)** | **CCH 22**  **(%, w/v)** | **SH 1**  **(%, w/v)** |
| --- | --- | --- | --- | --- | --- |
| **Ethanol titer after 72 h fermentation with GSE16-T18** | | | | | |
| **Unspiked hydrolysate** | 5.12  (100 %) | 4.11  (100 %) | 6.25  (100 %) | 3.96  (100 %) | 2.05  (100 %) |
| **0.3 g/L furfural** | - | 3.79  (92 %) | 5.73  (92 %) | 3.94  (99 %) | 1.99  (97 %) |
| **0.6 g/L furfural** | - | 3.78  (92 %) | 5.70  (91 %) | 3.98  (101 %) | 1.93  (94 %) |
| **1.0 g/L furfural** | - | 3.78  (92 %) | 4.55  (73 %) | 3.6  (91 %) | 1.80  (88 %) |
| **3.0 g/L furfural** | 3.91  (76 %) | 3.77  (92 %) | 1.31  (21 %) | 1.78  (45 %) | 0.63  (31 %) |
| **6.0 g/L furfural** | 2.84  (55 %) | 1.52  37 %) | 1.18  (19 %) | 1.20  (30 %) | 0.50  (24 %) |
| **Ethanol titer after 72 h fermentation with MD4** | | | | | |
| **Unspiked hydrolysate** | 5.05  (100 %) | 5.32  (100 %) | 6.29  (100 %) | 4.57  (100 %) | 2.67  (100 %) |
| **0.3 g/L furfural** | - | 5.18  (97 %) | 5.71  (91 %) | 3.33  (73 %) | 1.88  (70 %) |
| **0.6 g/L furfural** | - | 4.94  (93 %) | 5.12  (81 %) | 3.42  (75 %) | 1.70  (64 %) |
| **1.0 g/L furfural** | - | 4.94  (93 %) | 3.48  (55 %) | 3.19  (70 %) | 1.42  (53 %) |
| **3.0 g/L furfural** | 2.32  (46 %) | 2.51  (47 %) | 2.92  (46 %) | 3.02  (66 %) | 1.32  (49 %) |
| **6.0 g/L furfural** | 1.16  (23 %) | 1.01  (19 %) | 0.85  (14 %) | 0.54  (12 %) | 0.38  (14 %) |
